# Equol enhances tamoxifen’s anti-tumor activity by induction of caspase-mediated apoptosis in MCF-7 breast cancer cells

**DOI:** 10.1186/1471-2407-13-238

**Published:** 2013-05-15

**Authors:** Christiana Charalambous, Chara A Pitta, Andreas I Constantinou

**Affiliations:** 1 Department of Biological Sciences, University of Cyprus, 75 Kallipoleos str, PO box 20537, Lefkosia 1678, Cyprus

**Keywords:** Apoptosis, Breast cancer, Caspases, Equol, Tamoxifen

## Abstract

**Background:**

Soy phytoestrogens, such as daidzein and its metabolite equol, have been proposed to be responsible for the low breast cancer rate in Asian women. Since the majority of estrogen receptor positive breast cancer patients are treated with tamoxifen, the basic objective of this study is to determine whether equol enhances tamoxifen’s anti-tumor effect, and to identify the molecular mechanisms involved.

**Methods:**

For this purpose, we examined the individual and combined effects of equol and tamoxifen on the estrogen-dependent MCF-7 breast cancer cells using viability assays, annexin-V/PI staining, cell cycle and western blot analysis.

**Results:**

We found that equol (>50 μM) and 4-hydroxy-tamoxifen (4-OHT; >100 nM) significantly reduced the MCF-7 cell viability. Furthermore, the combination of equol (100 μM) and 4-OHT (10 μM) induced apoptosis more effectively than each compound alone. Subsequent treatment of MCF-7 cells with the pan-caspase inhibitor Z-VAD-FMK inhibited equol- and 4-OHT-mediated apoptosis, which was accompanied by PARP and α-fodrin cleavage, indicating that apoptosis is mainly caspase-mediated. These compounds also induced a marked reduction in the bcl-2:bax ratio, which was accompanied by caspase-9 and caspase-7 activation and cytochrome-c release to the cytosol. Taken together, these data support the notion that the combination of equol and tamoxifen activates the intrinsic apoptotic pathway more efficiently than each compound alone.

**Conclusions:**

Consequently, equol may be used therapeutically in combination treatments and clinical studies to enhance tamoxifen’s effect by providing additional protection against estrogen-responsive breast cancers.

## Background

Evidence from epidemiological studies suggest that nutrition plays an important role in the development of breast cancer, which remains the most common malignancy and the second most lethal cancer in women worldwide [1-4]. It was observed that the incidence of breast cancer is much lower in Asian women compared to Western women, and this was attributed to the daily consumption of soy products by Asian women, which contain phytoestrogens [5]. Equol (7-hydroxy-3-(4′-hydroxyphenyl)-chroman) is the bioactive metabolite of daidzein, a major phytoestrogen found in soy products. Recent studies suggest that equol has the greatest *in vitro* bioactivity and anti-oxidant activity when compared to soy isoflavones [6-8]. As known, 30-50% of the adult population cannot metabolize daidzein to equol and, interestingly, clinical response is usually limited to people who are “equol producers” [9,10]. Equol is reported to bind to both estrogen receptors ERα and ERβ, with a higher binding affinity for ERβ, which has been implicated in the inhibition of proliferation and induction of apoptosis in breast cancer cells [8,11-13]. Previous studies suggest that equol induces apoptosis in the ER negative breast cancer cells [14,15], while it seems to have a biphasic effect in ER-positive breast cancer cells enhancing cell proliferation at low concentrations (< 10 μM) [15-18] and possibly exerting an inhibitory effect at high concentrations (50–100 μM) [14]. As the role of equol in relation to breast cancer remains unclear, this study was designed to delineate the effect of equol on estrogen-dependent breast cancer cells using MCF-7 cells as a model system. This is particularly important as the controversy of results obtained in the soy isoflavone human intervention studies and the inability to establish the beneficial effects of soy isoflavones could be attributed to the failure to distinguish between “equol producers” and “non-equol producers” [10,19]. Therefore, the significance of evaluating the therapeutic potential of equol becomes more evident and may facilitate the design and implementation of future equol intervention studies for cancer.

Several reports suggest that equol and daidzein induce cell cycle arrest and apoptosis in breast cancer cells [2,8,14,20-25]. More specifically, it has been recently shown that daidzein induces MCF-7 breast cancer cell apoptosis via the intrinsic (mitochondrial) caspase-dependent apoptotic pathway [2]. However, the biological effects of equol have not been investigated as well as those of daidzein. Therefore, the aim of this study is to thoroughly explore the mechanism of equol-mediated apoptosis.

Tamoxifen, on the other hand, is an ERα antagonist classified as a non-steroidal selective estrogen receptor modulator (SERM), widely used in cancer chemoprevention and chemotherapy to prevent primary breast tumors or the development of recurrences, respectively [26-28]. Tamoxifen, and its bioactive metabolite 4-hydroxy-tamoxifen (4-OHT), inhibit proliferation and induce apoptosis in several types of ER-positive and ER-negative breast cancer cells, rat mammary tumors and other cancer types [29-34]. However, the anti-tumor mechanism of tamoxifen is not yet completely understood.

Accumulating experimental evidence from *in vivo* studies is beginning to support the possibility that soy components may enhance tamoxifen’s anti-tumor effect, by providing stronger protection against mammary carcinogenesis than tamoxifen alone [35,36]. Moreover, we have previously identified daidzein as the soy ingredient enhancing tamoxifen’s ability to prevent rat mammary tumor formation [37]. Since equol is the bioactive metabolite of daidzein [38,39], these findings support the premise that equol may potentiate tamoxifen’s efficacy against mammary carcinogenesis. We are reporting here the mechanism by which this daidzein metabolite enhances tamoxifen’s anti-tumor activity in ER positive breast cancer cells.

## Methods

### Cell culture

MCF-7 breast cancer cell line (obtained from ATCC) was cultured in MEM supplemented with 10% fetal bovine serum (FBS), 1% antibiotic-antimycotic, 1 mM sodium pyruvate, 1% non-essential aminoacids (MEM-NEAA), 2 mM L-glutamine (Gibco, Life Technologies, Paisley, UK) and 0.06 μg/ml insulin (Sigma, St. Louis, MI, USA). They were incubated at 37°C in a humidified incubator with 5% CO_2_. For estrogen deprivation, three days before treatment with equol or tamoxifen, cells were cultured in phenol-red free MEM supplemented with 10% dextran-coated charcoal (DCC) - treated FBS, 1% antibiotic-antimycotic, 1% non-essential aminoacids, 2 mM L-glutamine, 1 mM sodium pyruvate and 0.06 μg/ml insulin [40].

### Antibodies and reagents

Equol and 4-OHT were purchased from LC laboratories (Woburn, MA, USA) and Alexis Biochemicals (Enzo Life Sciences, Lausen, Switzerland), respectively. Reagents also included the pan-caspase inhibitor Z-VAD-FMK (Calbiochem, Nottingham, UK) and the MTT reagent (Sigma, St. Louis, MI, USA). The bcl-2, bax, glyceraldehyde 3-phosphate dehydrogenase (GAPDH) and cyclo-oxygenase-4 (COX-4) antibodies were purchased from Santa Cruz Biotechnology (Heidelberg, Germany) whereas the poly-(ADP ribose)-polymerase-1 (PARP-1), α-fodrin, caspase-9, caspase-8, caspase-7, caspase-6, cytochrome-c, and α-tubulin antibodies were purchased from Cell Signaling Technology (Danvers, MA, USA).

### MTT assay

The effect of equol, 4-OHT and their combination on MCF-7 viability was examined using the MTT (monotetrazolium) assay [41]. The cells were plated in 96-well plates (3×10^3^ cells/well) and treated with different concentrations of equol and 4-OHT for 24, 48 and 72 h. The MTT reagent was subsequently added (1:10 dilution) for 4 h at 37°C. The media were then removed and DMSO (150 μL/well) was added for 20 min. The absorbance, measured at 570 nm, was proportional to the number of viable cells per well.

### Mitochondrial/cytosolic extract preparation

Cells were cultured in 150-mm Petri dishes and treated for 48 h with vehicle control (DMSO and ethanol), equol (100 μM), 4-OHT (10 μM) or their combination. Mitochondrial and cytosolic extracts were prepared using the mitochondrial/cytosol fractionation kit (Abcam, UK).

### Western blot analysis

MCF-7 cells were treated with equol (100 μM), 4-OHT (10 μM) and their combination for 48 h, with or without Z-VAD-FMK (20 μM), and whole cell or mitochondrial/cytosolic extracts were prepared as previously described [42]. Protein content in the extracts was quantified using a bicinchoninic acid (BCA) protein assay kit (Pierce, Germany). Equal amounts of proteins (40 μg/lane) were separated on SDS-PAGE and electrotransferred to 0.45 μm nitrocellulose membranes. The membranes were then blocked with 5% non-fat dry milk in TBST (Tris buffered saline supplemented with 0.1% Tween-20) and probed with antibodies against PARP-1 (1:1000 dilution), α-fodrin (1:500 dilution), caspase-9 (1:500 dilution), caspase-8 (1:500 dilution), caspase-7 (1:500 dilution), caspase-6 (1:500 dilution), GAPDH (1:1000 dilution), bcl-2 (1:500 dilution), bax (1:500 dilution), COX-4 (1:250 dilution), cytochrome-c (1:250 dilution), and α-tubulin (1:1000 dilution) followed by HRP-conjugated anti-rabbit or anti-mouse immunoglobulin-G (IgG; 1:2000 dilution). Protein bands were detected by chemiluminescence using the Luminol substrate (Santa Cruz) according to the manufacturer’s protocol and analyzed using the UVP bioimaging system (Cambridge, UK).

### Cell death ELISA (Enzyme-linked immunosorbent assay)

MCF-7 cells were plated in 96-well plates in quadruplicates at a concentration of 3 × 10^4^ cells/ml (100 μl/well). Cells were treated with equol (100 μM), 4-OHT (10 μM) and their combination and lysed after 72 h. Lysates were analyzed for the presence of nucleosomes using the Cell Death Detection ELISA *Plu*s kit (Roche Diagnostics, Mannheim, Germany). Absorbance, measured at 405 nm, was proportional to cell death.

### Tali™ apoptosis kit

Cells were plated in 60-mm plates and treated with equol (100 μM), 4-OHT (10 μM) and their combination, with or without Z-VAD-FMK (20 μM). Cells were harvested 72 h post-treatment and stained using annexin-V Alexa Fluor® 488/PI (propidium iodide), as described by the Tali™ apoptosis kit (Life Technologies). Cell viability, death and apoptosis were evaluated using the Tali™ Image-based Cytometer (Life Technologies). The annexin-V positive/PI negative cells were recognized as apoptotic cells by the cytometer software whereas the annexin V positive/PI positive cells were identified as dead cells. Similarly, the annexin V-negative/PI negative cells were identified as viable cells.

### Cell cycle analysis

Cells were plated in 100-mm plates and treated with equol (100 μM), 4-OHT (10 μM), and their combination for 6, 12, 24, 48 and 72 h. They were harvested, fixed in 70% ethanol, incubated with the PI staining solution (containing 1 mg/ml PI and 100 μg/ml RNase) for 15 min at 37°C and analyzed for DNA content using the Guava EasyCyte™ flow cytometer and the GuavaSoft analysis software (Millipore, Watford,UK).

### Statistical analysis

Values are presented as the mean ± SEM. Statistical significance was evaluated using student’s t-test for paired comparison. *P<0.05* was considered statistically significant. Data are representative of three individual experiments. Each experimental group was repeated in triplicates or quadruplicates, as described in the Figure Legends section.

## Results

### Equol and 4-OHT reduce MCF-7 viability

**Figure 1:**
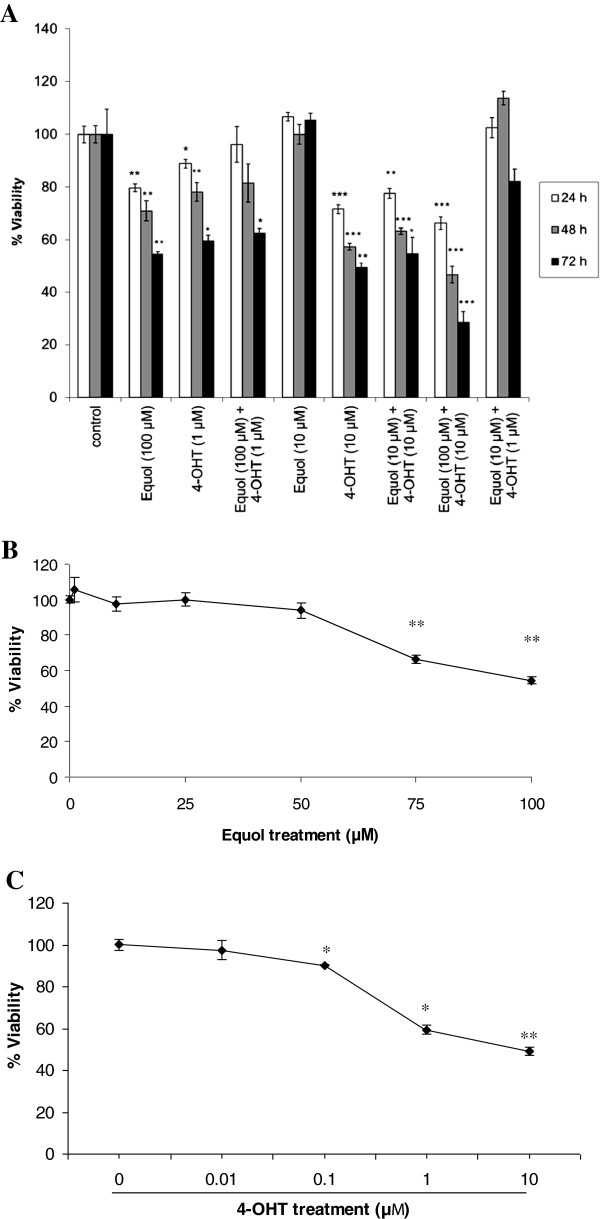
**Comparison of the effect of equol and 4-OHT on MCF-7 cell viability.** (**A**) Cells (3 × 10^3^/well) were plated in 96-well plates and treated with different concentrations of equol and 4-OHT, individually or combined. After 24, 48 and 72 h, cell viability was evaluated using the MTT assay. The OD reading at 570 nm was proportional to cell viability. * P < 0.05, ** P < 0.005 and *** P <0.0005. *P*_*Equol (100 μM)*__*vs control*_*= 0.003; P*_*4-OHT(10 μM)*__*vs control*_*= 0.002*; *P[*_*Equol (100 μM)+4-OHT (10 μM)] vs control*_*= 0.0003*. Dose response curves for equol (**B**) or 4-OHT (**C**) effect on MCF-7 cell viability. Cells (3 × 10^3^/well) were seeded in 96-well plates and treated with different concentrations of equol (**B**) or 4-OHT (**C**). After 72 h, cell viability was evaluated using the MTT assay. The data are expressed as percentage change in viability in comparison to the vehicle treated control group. Each experimental group was repeated in quadruplicates and data are representative of three individual experiments. Bars correspond to the standard error of mean (SEM).

To examine the ability of equol and 4-OHT to inhibit MCF-7 cell growth, their individual and combined effects on cell viability were observed. Equol (> 50 μM) and 4-OHT (>100 nM) provoked a marked reduction in MCF-7 viability in a dose- and time-dependent manner (Figure 1A-C). In contrast, lower concentrations of equol (1 nM- 1 μM) did not exert a significant effect on cell growth (data not shown). Futhermore, the combination of equol (100 μM) and 4-OHT (10 μM) reduced cell viability in an additive manner (72 h; Figure 1A), suggesting that equol enhances tamoxifen’s anti-proliferative effect in MCF-7 cells.

### Equol and 4-OHT induce MCF-7 cell death via apoptosis

**Figure 2:**
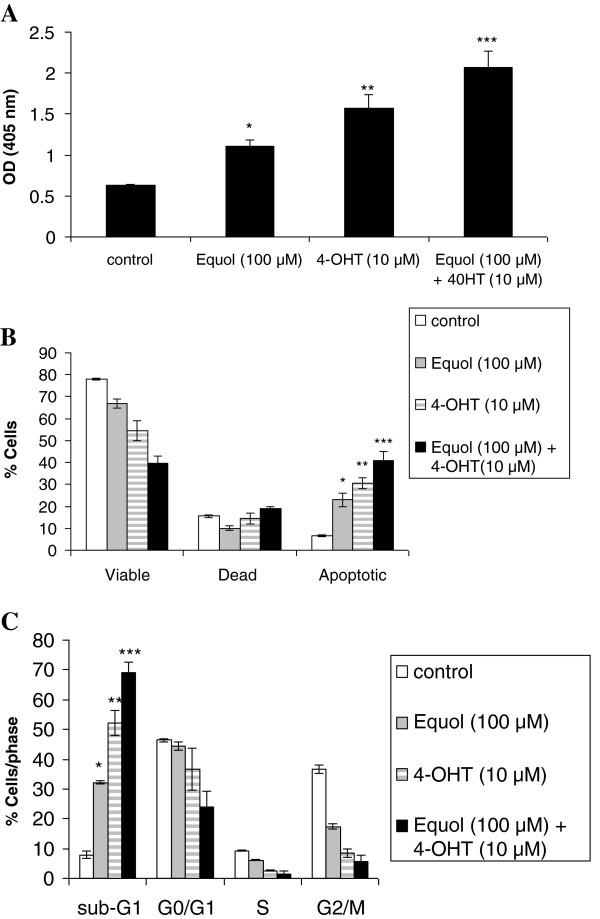
**Effect of equol and 4-OHT on cell death (A), apoptosis (B) and cell cycle distribution (C).** For the determination of cell death (**A**), MCF-7 cells were seeded in 96-well plates (3 × 10^3^ cells/well). Upon attachment cells were treated with equol (100 μM) and/or 4-OHT (100 μM). After 72 hours, cell death was evaluated using the Cell Death ELISA. The OD reading at 405 nm was proportional to the number of nucleosomes released in the cell lysates of the cells. The data are expressed as OD (405 nm) in comparison to the vehicle- treated control group. Each group was repeated in quadruplicates.* P_*Equol vs control*_*= 0.023*; ** *P*_*4-OHT vs control*_*= 0.032*; *** *P*_*[Equol+4-OHT] vs control*_*= 0.016*. (**B**) Effect of equol and 4-OHT on MCF-7 cell apoptosis using annexin-V Alexa Fluor® 488/PI staining. Cells were plated in 60-mm plates and treated with equol (100 μM) and 4-OHT (10 μM) for 72 h. Cell viability, death and apoptosis were evaluated using the Tali™ apoptosis kit and the Tali™ Image-based Cytometer. Each experimental group was repeated in triplicate. Bars correspond to the standard error of mean (SEM). * *P*_*Equol vs control*_*=0.032*; ** *P*_*4-OHT vs control*_*=0.011*; *** *P*_*[Equol + 4-OHT] vs control*_*= 0.013*. (**C**) Effect of equol and 4-OHT on cell cycle distribution using PI staining. MCF-7 cells were treated with equol (100 μM) and 4-OHT (10 μM) for 72 h. Cell cycle distribution was evaluated using PI staining for 15 min at 37°C. Sample analysis was performed using the Guava EasyCyte™ flow cytometer and the GuavaSoft analysis software. Each experimental group was repeated in triplicate. Bars correspond to the standard error of mean (SEM). * *P*_*Equol vs control*_*= 0.0025*; ** *P*_*4-OHT vs control*_*= 0.026*; *** *P*_*[Equol+4-OHT] vs control*_*= 0.0037.*

We began evaluating the mechanism implicated in the reduction of MCF-7 cell viability by determining cell death following treatment with equol and 4-OHT. These compounds induced MCF-7 death after 72 h of treatment (Figure 2A). Interestingly, their combination enhanced cell death in an additive manner (*P*_*[Equol+4-OHT] vs. 4-OHT*_ *= 0.028; P*_*[Equol+4-OHT] vs. Equol*_*=0.023*).

To examine whether cell death was mediated through apoptosis, cells were stained with annexin-V/PI following treatment with equol and 4-OHT. Each compound produced a substantial increase in the percentage of apoptotic cells (Figure 2B). The combination of equol and 4-OHT had an additive effect on cell apoptosis (*P*_*[Equol+4-OHT] vs. 4-OHT=*_*0.028; P*_*[Equol+4-OHT] vs. Equol*_ *= 0.018*).

The effects of equol and tamoxifen on cell cycle progression were also determined using flow cytometry. Even though no substantial changes were evident in cell cycle distribution from 6–48 h of treatment (data not shown), significant increase in the sub-G_1_ phase, which is indicative of apoptosis, was observed at 72 h, accompanied by a marked reduction in the percentage of cells in the G_0_/G_1_, S and G_2_/M phases (Figure 2C). These results show that 68.9±3.6% of the cells treated with the equol/4-OHT combination were in the sub-G_1_ phase, which is significantly higher than the corresponding percentage of equol-treated cells (32.1±0.5%), 4-OHT-treated cells (52.1±4.2%) or vehicle control treated cells (7.8±1.1%) (*P = 0.0037*; Figure 2C). Taken together, these results indicate that these agents do not induce cell cycle arrest, and that their combination is more effective in activating apoptosis than each compound alone. This is consistent with our previous data, demonstrating that equol and 4-OHT do not increase p53 and p21 expression, which is up-regulated in cells undergoing G_1_ arrest (data not shown).

### Z-VAD-FMK inhibits equol and 4-OHT mediated apoptosis

**Figure 3:**
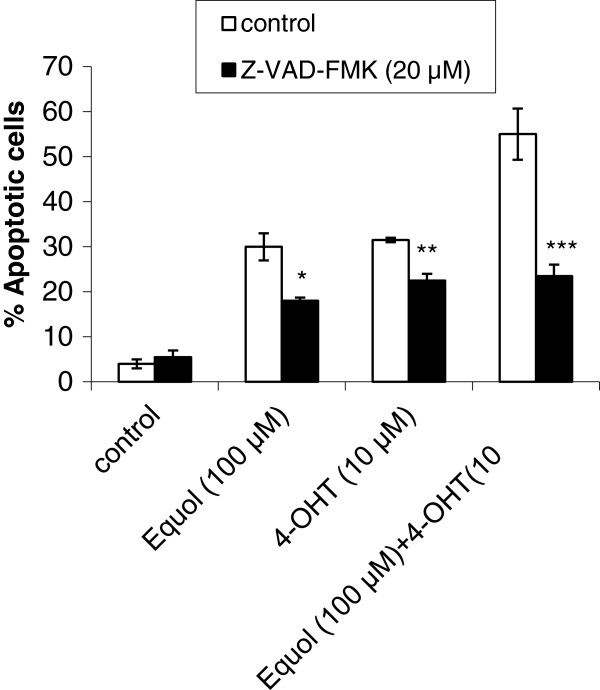
**Effect of the pan-caspase inhibitor Z-VAD-FMK on equol and 4-OHT induced MCF-7 cell apoptosis.** Cells were plated in 60-mm plates and treated with equol (100 μM) and 4-OHT (10 μM) for 72 h. Cell apoptosis was evaluated using annexin-V Alexa Fluor® 488/PI staining and the Tali™ Image-based Cytometer. Each experimental group was repeated in triplicates and data are representative of three individual experiments. The bars correspond to the SEM. * *P*_*Equol vs [Z-VAD-FMK+Equol]*_ *= 0.014*; ** *P*_*4-OHT vs [ZVAD-FMK+4-OHT]*_*=0.012*; *** *P*_*[Equol+4OHT] vs [Z-VAD-FMK+Equol+4-OHT]*_*=0.017.*

To elucidate the precise pathways involved in equol- and 4-OHT-induced apoptosis, cells were treated with the pan-caspase inhibitor Z-VAD-FMK in combination with equol and/or 4-OHT and apoptosis was evaluated using annexin-V/PI staining. Z-VAD-FMK significantly inhibited equol- and 4-OHT-induced apoptosis, indicating activation of the caspase-dependent pathway by these compounds (Figure 3). However, the inhibition was not complete, suggesting that caspase-independent mechanisms may be implicated in addition to the caspase dependent mechanisms.

### Equol and 4-OHT induce PARP and α-fodrin proteolysis

**Figure 4:**
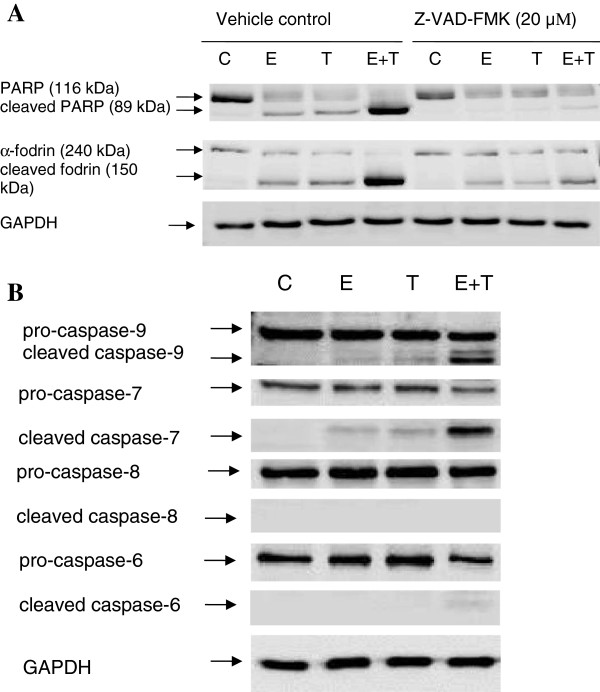
**Effect of equol and 4-OHT on pro-apoptotic protein (A) and caspase (B) expression.** MCF-7 were treated for 48 h with equol (100 μM) and 4-OHT (10 μM) with and without Z-VAD-FMK (20 μM) (**A**), or without Z-VAD-FMK (**B**), and whole cell extracts were prepared. Protein expression was then analyzed by western blot. Data are representative of three individual experiments. C, vehicle control; E, Equol (100 μM); T, 4-OHT (10 μM), E + T, Equol (100 μM) + 4-OHT (10 μM).

The apoptotic mechanisms involved in the death response to equol and 4-OHT were further characterized by monitoring PARP and α-fodrin expression using western blotting. PARP and α-fodrin are known substrates cleaved by the effector caspases-3 and −7, which are activated in apoptotic cells [43,44]. PARP and α-fodrin proteolysis was evident with equol or 4-OHT treatment and was significantly enhanced by their combination (Figure 4A). This effect was prevented to a large extent by Z-VAD-FMK (Figure 4A), reconfirming that equol- and 4-OHT activate caspase-mediated apoptosis.

### Equol and 4-OHT induce apoptosis via the intrinsic pathway

Based on our previous results suggesting activation of caspase-dependent apoptosis by equol and 4-OHT, we examined their effect on caspase expression and activation. To distinguish between the intrinsic and the extrinsic apoptotic pathways, we investigated the effect of equol and 4-OHT on the initiator caspases −8 and −9 and the effector caspases −6 and −7. Equol and 4-OHT induced a pronounced pro-caspase-7 and pro-caspase-9 cleavage and activation, which was greatly enhanced by their combination (Figure 4B). In contrast, caspase-8 and caspase-6 remained unaffected by these treatments (Figure 4B), indicating that these compounds act mainly through the intrinsic apoptotic pathway.

### The combination of equol and 4-OHT promotes cytochrome-c release and reduction of bcl-2 expression

**Figure 5:**
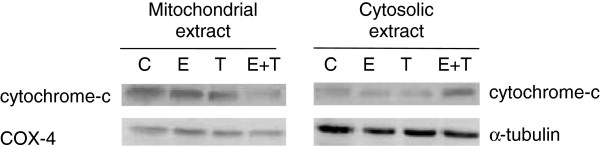
**Effect of equol and 4-OHT on cytochrome-c expression.** MCF-7 were treated for 48 h with equol (100 μM) and 4-OHT (10 μM) and mitochondrial and cytosolic extracts were prepared. Protein expression was then analyzed by western blot. Data are representative of three individual experiments. C, vehicle control; E, Equol (100 μM); T, 4-OHT (10 μM), E + T, Equol (100 μM) + 4-OHT (10 μM).

The key event causing caspase-9 cleavage, and thus activation of the intrinsic apoptotic pathway, is cytochrome-c release from the mitochondria to the cytosol [45]. Therefore, we explored the effect of equol and tamoxifen on cytochrome-c expression and localization. The combination of equol and 4-OHT induced a substantial cytochrome-c release from the mitochondria to the cytosol of MCF-7 cells (Figure 5) which was not detected in cells treated with equol or 4-OHT alone, thus confirming the activation of the intrinsic apoptotic pathway.

**Figure 6:**
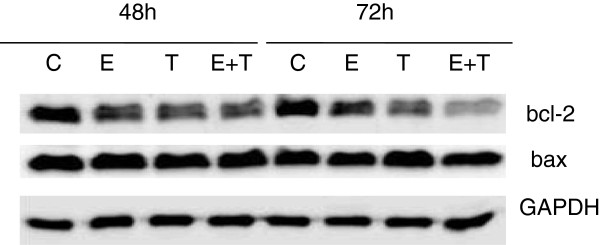
**Effect of equol and 4-OHT on bcl-2 and bax expression.** MCF-7 were treated for 48 and 72 h with equol (100 μM) and 4-OHT (10 μM) and whole cell extracts were prepared. Protein expression was then analyzed by western blot using anti-bcl-2 and anti-bax polyclonal antibodies. Data are representative of three individual experiments. C, vehicle control; E, Equol (100 μM); T, 4-OHT (10 μM), E + T, Equol (100 μM) + 4-OHT (10 μM).

To complete the picture, we investigated the effect of the two compounds and their combination on the expression of the anti-apoptotic protein bcl-2 and the pro-apoptotic protein bax [46]. Bcl-2 and bax are proteins that can prevent or facilitate cytochrome-c release from the mitochondria respectively, thus inhibiting or promoting apoptosis [47]. The bcl-2:bax ratio is important in determining whether a cell will undergo apoptosis or survive [47]. We found that equol and 4-OHT induced a time-dependent reduction in the total levels of bcl-2 in MCF-7 cells, whereas they did not affect bax expression (Figure 6). The combination of equol and tamoxifen had an additive effect in the reduction of bcl-2 expression, which was more evident at 72 h (Figure 6). Equol and 4-OHT did not affect bcl-2 or bax expression at 24 h of treatment (data not shown). Therefore, equol and 4-OHT induce a time-dependent reduction of the bcl-2:bax ratio, promoting in this way cytochrome-c release and activation of the intrinsic apoptotic pathway.

## Discussion

In this study, we evaluated the individual and combined effects of equol and 4-OHT, the bioactive metabolite of tamoxifen, in the ER positive MCF-7 breast cancer cells. Our findings show for the first time that equol not only does not abolish the anti-tumor effects of tamoxifen, but instead it induces apoptosis and significantly enhances tamoxifen’s pro-apoptotic effects in these cells (Figure 1A-C and Figure 2A-C). Moreover, the pan-caspase inhibitor Z-VAD-FMK significantly inhibited equol- and tamoxifen- induced apoptosis (Figure 3), suggesting that these compounds activate the caspase-mediated apoptotic pathway. However, the inhibition was not complete, suggesting that caspase-independent mechanisms may also be involved in equol and tamoxifen induced apoptosis. Previous studies support our findings showing that equol inhibits MCF-7 proliferation and induces caspase-mediated apoptosis in ER negative breast cancer cells and rat mammary tumors [8,48,49]. With respect to tamoxifen, previous studies provide evidence that tamoxifen induces caspase-dependent apoptosis in MCF-7 and other types of cancer cells [30,32,50-53]. Even though high concentrations of equol (100 μM) were required to activate MCF-7 apoptosis, which are not physiologically achievable in human plasma due to metabolic conversion of the active aglycone equol to the inactive conjugated form [54], our results may find applications in targeted immunotherapies, which may enable maximal delivery of equol into the cancer cells. This strategy was previously used successfully for genistein, which was immunoconjugated with a monoclonal antibody and targeted to a B cell-specific receptor for treatment of an animal model of B-cell precursor leukemia [55].

To fully explore the apoptotic pathway activated by equol and tamoxifen, we investigated their effects on key proteins involved in apoptosis, such PARP, α-fodrin and caspases −6, -7, -8 and −9. Caspase-9 is part of the intrinsic (mitochondrial) apoptotic pathway and is activated by cytochrome-c release from the mitochondria, whereas caspase-8 is part of the extrinsic apoptotic pathway activated by external signals through the death receptors [45]. Active caspase −9 and caspase-8 in turn induce cleavage and activation of the effector caspases −3, -6 and −7 [43,45,56,57], which subsequently cleave nuclear and cytosolic targets, such as PARP and α-fodrin, resulting in cell destruction [43,44]. Since MCF-7 cells are deficient of functional caspase-3, the effector caspase-7 is responsible for apoptosis in these cells [58-60]. Our experiments show that equol and 4-OHT induce PARP and α-fodrin proteolysis, which was significantly enhanced by their combination and partially inhibited by the pan-caspase inhibitor Z-VAD-FMK (Figure 4A), suggesting that additional proteases besides caspases may be involved in equol- and tamoxifen-induced apoptosis. Furthermore, the combination of equol and tamoxifen induced a pronounced caspase-9 and caspase-7 cleavage accompanied with cytochrome-c release into the cytosol, without affecting caspases-6 and −8 (Figure 4B and Figure 5). Treatment with either equol or tamoxifen, on the other hand, had a lesser effect on caspase-9 and caspase-7 cleavage associated with a trivial effect on cytochrome-c release from the mitochondria into the cytosol. Consequently, the combination of equol and tamoxifen is significantly more potent in inducing MCF-7 cell apoptosis than each compound alone. Therefore, our data suggest that equol and tamoxifen activate the intrinsic apoptotic pathway. Previous studies support our findings as they have shown activation of the intrinsic apoptotic pathway in MCF-7 cells by tamoxifen and daidzein [2,29-31,51,61,62]. Moreover, equol and tamoxifen induced a time-dependent reduction in blc-2 expression and hence the bcl-2:bax ratio, which was further reduced by the combination of the two compounds (Figure 6). Decreased bcl-2 expression was observed in several cancer cell types treated with tamoxifen and daidzein [14,63,64] and in equol-induced apoptosis in mammary carcinomas [14,48].

## Conclusions

In conclusion, this study suggests that equol induces MCF-7 cell apoptosis and enhances tamoxifen’s pro-apoptotic effect via activation of the intrinsic apoptotic pathway. The significance of our findings is that women with ER-positive early-stage breast cancer, undergoing tamoxifen adjuvant treatment, may be further benefitted by co-treatment with pharmacological doses of equol. Our results also suggest that “equol producers” may be at lower risk of developing breast cancer due to the apoptotic action of equol against ER positive breast cancer cells. Future clinical trials designed to determine the safety and efficacy of equol in adjuvant hormonal therapy against breast cancer are warranted.

## Abbreviations

BCA: Bicinchronic acid; COX-4: Cyclo-oxygenase-4; DMBA: 6,12 - dimethylbenz[a]anthracene; ELISA: Enzyme-linked immunosorbent assay; ER: Estrogen receptor; FBS: Fetal bovine serum; GAPDH: Glyceraldehyde 3-phosphate dehydrogenase; IgG: Immunoglobulin G; MTT: Monotetrazolium; 4-OHT: 4-hydroxy-tamoxifen; PARP: Poly (ADP ribose) polymerase; PI: Propidium iodide; SERM: Selective estrogen receptor modulator.

## Competing interests

The authors declare that they have no competing interests.

## Authors’ contributions

CC carried out all the experiments included in this manuscript and participated in the design, data acquisition, analysis and interpretation. CAP provided assistance with some of the experiments and valuable feedback. AIC participated in the experimental design, data analysis and interpretation. Both CC and AIC participated in drafting and critically revising the manuscript. All authors read and approved the final manuscript.

## Pre-publication history

The pre-publication history for this paper can be accessed here:

http://www.biomedcentral.com/1471-2407/13/238/prepub

## Study design

The role of equol, tamoxifen and their combination in breast cancer treatment.
